# The use of smartphone apps in clinical practice: A survey of South African physiotherapists

**DOI:** 10.4102/sajp.v76i1.1327

**Published:** 2020-04-20

**Authors:** Michael Rowe, Berenice Sauls

**Affiliations:** 1 Department of Physiotherapy, University of the Western Cape, Cape Town, South Africa

**Keywords:** health-related apps, medical apps, mobile apps, smartphone apps, clinical practice

## Abstract

**Background:**

There is anecdotal evidence that physiotherapy clinicians increasingly are using medical apps and health-related apps as part of their clinical practice, and in some cases, even ‘prescribing apps’ to patients. However, there is limited information on how South African physiotherapists use, and what they think about, the integration of mobile apps in their practice.

**Objectives:**

This study aimed to describe the use of smartphone apps as part of clinical practice in a small group of South African physiotherapists.

**Method:**

This study made use of a cross-sectional, descriptive design and a self-administered questionnaire to survey practitioners. The population included all 1300 physiotherapists who were registered with the Orthopaedic Manipulative Physiotherapists Group (OMPTG) of the special interest group of the South African Society of Physiotherapists (SASP), with a sample of 270 out of 1300 who responded (response rate = 21%). Descriptive data are presented using graphs, figures and percentages, and responses to open-ended questions are included in support of the themes.

**Results:**

The majority of the participants used apps as part of their practice (60%) but did not prescribe apps to patients. Most apps are used for administrative and communication purposes. Even clinicians who used apps themselves expressed concerns about prescribing them to patients, and there were clear misconceptions about the use of apps.

**Conclusion:**

Many clinicians in this study reported that there were real benefits to the use of smartphone apps as part of their practice. However, they raised concerns about the more general prescription of apps for clients.

**Clinical implications:**

Further research, education and collaboration amongst all stakeholders is necessary to produce guidelines for the use of apps in clinical practice

## Background

In 2017, there were more than 325 000 health-related and medical apps in the app stores of all major mobile operating systems, with some of these apps having been downloaded hundreds of thousands of times (Pohl 2017). In the context of smartphones, ‘apps’ describe mobile applications designed to provide extra functionality for users, including browsing the web, listening to audio, watching video and creating new content (Ravenek & Alvarez 2016). Twenty per cent of American smartphone users report having downloaded a health-related app, the most popular of which are used to monitor exercise, diet and weight (Lupton 2014). Following this trend, the medical literature now refers to the practice of ‘prescribing apps’ to patients who use these apps to monitor their activity and use the resulting data to change their behaviours with the aim of reducing the risks associated with their conditions (Brustein 2012). Mobile apps are expected to play an increasingly important role in health care, where patients gather personal data that can be used – either by themselves, or in conjunction with health care providers – to help them make informed choices about their health. These data can also be shared with health care providers and funders to support decision-making at higher levels in the health system (Aitken 2013).

There is a pattern that shows clinicians using apps more frequently at the bedside, citing an increase in efficiency by saving time and allowing more rapid decision-making at the point of care (Lindquist et al. 2008; Prgomet, Georgiou & Westbrook 2009). However, there are concerns about how patients may respond to clinicians’ use of a smartphone during a clinical encounter and the potential negative impact on their communication. For example, there is a concern that clinicians may become distracted with social media notifications while looking up relevant information on the patient’s condition (McAlearney, Schweikhart & Medow 2004). There are other important factors to take into account when considering the use of mobile apps in clinical practice. Regardless of whether an app has been designed for use by a health care professional or a member of the public, it is available for download by anyone. Thus, health-related apps have become a business opportunity for investors, opening up a new sector in health care into which software developers, marketers and health care professionals are moving (Pohl 2017). It is also possible that consumers may choose to download the most popular apps, which is not always a good indicator of quality or accuracy (Aitken 2013).

A more specific example from physiotherapy clinical practice is the use of goniometer apps, which have been found, in some circumstances, to be more reliable than the universal goniometer (Milanese et al. 2014). While these developments have the potential to change how physiotherapists practise, there are some challenges with the use of apps in clinical practice, not least of which is the high cost of a smartphone relative to universal goniometers. In addition, these apps must often be used in very specific ways because the software makes certain assumptions about the ‘typical’ adult who has predictable anatomical ratios (Milanese et al. 2014). Therefore, the accuracy and utility of these apps must be interpreted within a narrow context and with significant limitations, not all of which will be evident to inexperienced clinicians.

Medical apps and health-related apps are rapidly increasing in number and scope, and many of them aim to provide medical and health-related information for both professionals and consumers. Attempts to regulate the use of these apps are in a nascent stage, meaning that there is little guidance for physiotherapists and physiotherapy students who either currently use, or are considering using, apps as part of their professional practice. In order to make effective use of mobile apps in health care, while avoiding the negative implications, there is a need to understand the use of apps by physiotherapists in a South African context.

This study, therefore, aimed to describe South African physiotherapists’ use of smartphone apps as part of their professional practice.

## Method

The cross-sectional, descriptive design aimed to provide a profile of use of smartphone apps amongst a limited sample of South African physiotherapists. A survey was used to gather data on the kinds of real-world use of apps that are currently lacking in the South African physiotherapy context. A self-administered questionnaire was developed using the available literature (Aitken 2013; Lindquist et al. 2008) to support the objectives of our study. The questionnaire included closed-ended questions that aimed to identify how physiotherapists make use of smartphone apps as part of their clinical practice as well as open-ended questions to explore their experiences around the use of apps.

The questionnaire consisted of a series of Yes/No responses and multiple choice questions. The survey was piloted amongst 12 members of the Orthopaedic Manipulative Physiotherapists Group (OMPTG), a special interest group of the South African Society of Physiotherapy (SASP), in order to improve the face value of the questions, as well as to improve clarity and remove ambiguity. Minor changes were made to the questions based on the responses of these participants, who were excluded from the final survey. The questionnaire was implemented using Google Forms, and all responses were collected online.

The link to the online survey was sent to the Chairperson of the OMPTG group of the SASP who distributed it to their national mailing list. An information sheet was included with the questionnaire informing participants (*N* = 1300) of the purpose of the study, as well as their right to withdraw at any stage of the study. A reminder email was sent 2 weeks later, and a final reminder was sent 2 weeks after that. No incentives were offered to participants who completed the survey. No personally identifiable information was gathered and all participants were anonymous.

Descriptive data are presented using graphs, figures and percentages, and responses to open-ended questions were analysed with inductive content analysis using Atlas.ti. Inductive analysis consists of three phases, namely: preparation, organising and reporting (Elo & Kyngäs 2008).

Within the organising phase, the authors read and re-read the data to familiarise themselves with the data before coding was initiated. Codes were further grouped and categorised into themes. After revisiting the codes and themes, a report was developed, which described the themes with substantiating quotes, presented in a tabulated form.

### Ethical consideration

The study received ethical clearance from the University of the Western Cape Research Ethics Committee (registration number: 15/6/79).

## Results

### Demographics

**FIGURE 1:**
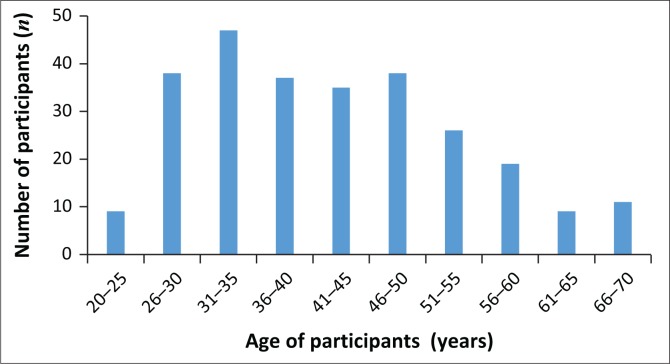
Ages of participants.

A total of 270 participants completed the survey, resulting in a response rate of 21%. The majority of participants were female (*n* = 248; 92%). Figure 1 presents the wide age distribution of participants.

### Use of apps in professional practice

Of the 270 participants, 14 (5%) participants did not have a smartphone; therefore, they did not complete the remainder of the questionnaire, and one participant who reported having a smartphone chose not to continue. The number of participants who, therefore, completed the full survey was 255 (20%).

**FIGURE 2:**
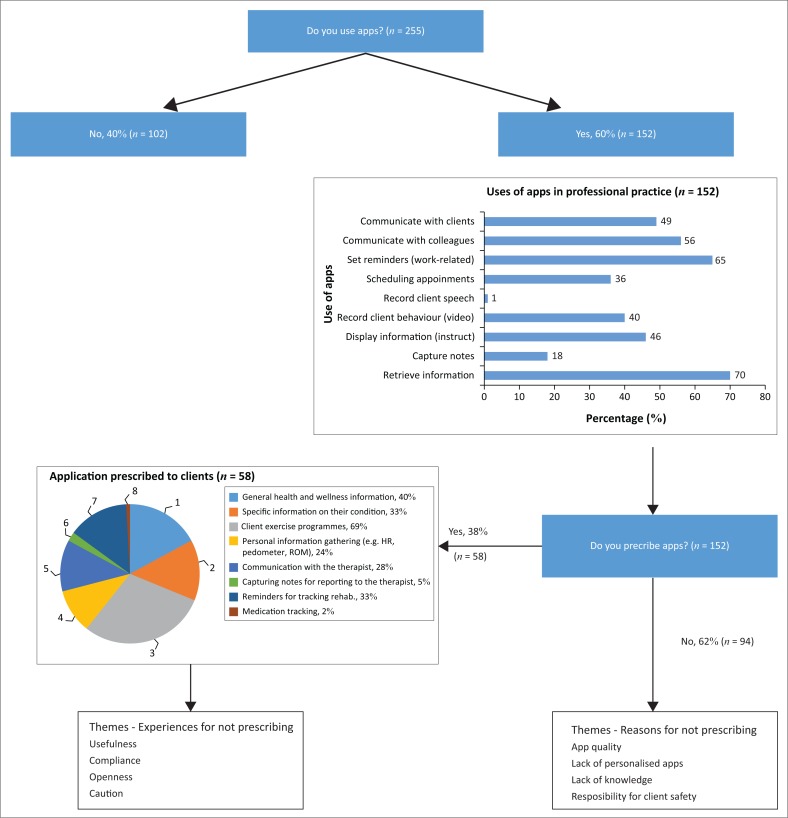
Breakdown of participant responses to the survey.

Figure 2 presents a flow diagram of the most important findings of the study.

Of the participants who had smartphones at the time of the study (*n* = 255; 94%), 152 (60%) reported using apps as part of their professional practice. These participants reported using apps for administrative purposes, for example, setting reminders and communicating with colleagues and patients. Of the 152 participants who reported using apps in practice, 58 (38%) had prescribed medical or health-related apps to their clients, with apps for exercise programmes being the most commonly prescribed ones. The next most commonly prescribed apps were those used to provide information on general health and wellness, and the apps for setting reminders and tracking rehabilitation programmes.

Those participants who reported that they had prescribed apps to clients (*n* = 58; 38%) were then asked to respond to the open-ended question, *Can you please provide some more detailed information about the experience of prescribing an app to a client?* The responses were categorised into the following themes: usefulness, compliance, openness and caution. Themes and participant responses are presented in Table 1.

**TABLE 1:** Participants who prescribed apps to clients.

Theme	Participant responses
**Usefulness** Participants noted that their clients had reported that the prescribed apps were useful, helpful and generally reported the use of apps as a positive experience.	‘Patients [*clients*] found it useful. I would definitely recommend using [*apps*]’ ‘…client found it helpful’ ‘I always get positive feedback after prescribing an app to patients [*clients*]’
**Compliance** Participants reported using apps to help clients and improve compliance, as they helped to set goals, and some were also able to record some aspects of client activity.	‘They [*apps*] seem to improve patient compliance’ ‘…set a goal as to doing it [*exercise*] three times a week’ ‘…encourage patients [*clients]* to move daily and to measure the distances, walked or ran.’
**Openness** Participants reported that younger and more technologically literate clients were more receptive to having apps prescribed to them.	‘Younger patients [*clients*] are more receptive to using apps.’ ‘Patients [*clients*] are all enthusiastic but it is only ones really into using technology that keep on using their apps’ ‘Some patients [*clients*] who are very tech savvy, enjoy [using apps] and their rehabilitation is enhanced by the use of apps’
**Caution** Practitioners highlighted the need for reviewing apps and using the apps themselves before prescribing them to clients.	‘…important to revise the app first’ ‘I always do [*the exercises on app*] myself before suggesting to a patient [*client*]’ ‘Go into the app and look through [*review*] the exercises’

The majority of participants reported that they did not prescribe apps to patients as part of their management programme (*n* = 94; 62%). Their reasons for this were categorised into app quality, lack of personalisation, lack of knowledge and responsibility for patient safety. Themes and participant responses are presented in Table 2.

**TABLE 2:** Reasons for not prescribing apps.

Theme	Participant responses
**App quality** Some participants were concerned with the accuracy and usefulness of apps and online information in general.	‘I seldom find information on apps and internet sites accurate or relevant to the patients’ [*clients*’] condition…’ ‘I haven’t found any of the apps useful’ ‘No appropriate app, prefer to give specific instruction on a case by case basis’
**Lack of personalised apps** Participants highlighted the need for individualised, patient-specific apps and believed that a general app would not provide appropriate, specific information.	‘Not specific enough for the individual patient’ ‘…as each patient has a unique presentation and condition’ ‘I think the personal touch of therapy is lost and is treated as the average person should be able to do this and this’
**Lack of knowledge** There was a lack of knowledge amongst participants as to what appropriate apps were available to use.	‘Need some teaching and guidance’ ‘I haven’t done enough research on apps that the contents good enough’ ‘haven’t really looked for one’
**Responsibility for client safety** Concerns were raised by the practitioner with regard to the legality and responsibility of patients’ safety and if the use of an app would lead to injury or regression in the management of the patient.	‘I wonder about the legality and who takes responsibility should something go wrong’ ‘…could potentially be misleading or harmful to a patient [*client*]’ ‘I don’t want the patient [*client*] to find information that is incorrect [*on apps*]’

## Discussion

The dominant use of apps by participants in this study was for administrative purposes in clinical practice. More than half of the participants in this study used apps as part of their own professional practice although only a small number actually prescribed apps to their patients. The most commonly reported purpose for using apps was to retrieve information, followed by the use of reminders and communication apps that were used to interact with colleagues and/or patients. These findings are similar to what has been reported in the literature, where apps for reminders and communication have been used to improve patient compliance and appointment attendance (Kassianos et al. 2017; Schwebel & Larimer 2018). There is evidence that the use of appointment reminders can reduce patients’ non-attendance, which has led to improved outcomes (Moran, O’Loughlin & Kelly 2018).

Communication apps were the second most frequently used apps in participants’ professional practice, which is again similar to studies showing that apps are predominantly used for communication purposes (Ventola 2014). This suggests that patients use communication apps to improve communication about their health status with their service providers (Jamison et al. 2017), although it is unclear what therapeutic effect this might have on their rehabilitation. This enhanced communication between patients and service providers can be synchronised and shared, thus enabling more efficient communication, which may include better feedback, more timely recommendations and improved support for patients, all of which may result in better services (Abelson et al. 2017; Chen et al. 2017a). There is thus some evidence that integrating apps for communication may be an appropriate use of ‘regular’ smartphone apps (i.e. apps not specifically designed for health-related uses). However, as participants in this study also noted, there are concerns about patient privacy, including the transmission of personal information over channels of communication that are not controlled either by the service provider or the patient. There is thus a need for health care professionals to ensure that they take steps to ensure that patient information is protected, for example, by using communication apps – like WhatsApp or Signal – that include data encryption by default.

Many participants reported that they did not feel comfortable prescribing apps to patients for their personal use, indicating that they may not have realised that the administrative apps they use could be beneficial for patients. Participants who had prescribed apps to patients found them to be useful as they reported that it had improved their compliance when they were used for goal-setting or for sending reminders with respect to exercise programmes or upcoming appointments. In addition, there is some evidence that health-related apps can improve health and feelings of self-reliance in patients (Anderson, Burford & Emmerton 2016), and they can also enhance patient-centred care by improving access to health information (Baldwin et al. 2017). Patients may also demonstrate increased adherence to the programme when using health-related apps (Jamison et al. 2017). Even though some participants in this study reported the benefits of prescribing apps to patients, a more holistic approach is required to achieve common goals, such as improved clinical decision-making, improved accuracy, increased efficiency and enhanced productivity (Ventola 2014). It may be that professional organisations and governmental institutions will need to be involved in the process in order to ensure that both clinicians and patients are protected.

Participants who did not prescribe apps reported that they were concerned about app quality, personalisation of apps, patient safety and their own lack of knowledge of the apps. The incorporation of apps in practice is a fairly new concept in health care and some may, therefore, be reluctant to make use of apps in practice (Ravenek & Alvarez 2016). It is important to recognise that the reasons participants in this study gave for not prescribing apps are legitimate and were informed by concerns driven by limitations in the knowledge of the clinician, as well as perceptions of how the use of apps may impact on the therapist–patient relationship. Health professionals need to be aware of these challenges before making recommendations about the use of apps to patients (Aitken 2013). Without oversight and regulation, there may be a risk that unqualified stakeholders, for example, software developers and venture capitalists, may influence the direction of patient and professional decision-making in a clinical context. In addition, as the number of apps proliferates, there is a concern that it will lead to app overload, making the selection of appropriate apps potentially overwhelming for both clinicians and patients, as they find it increasingly difficult to differentiate between useful apps and those that are potentially dangerous (Aitken 2013).

Participants raised concerns about whether the information provided by apps could be trusted, highlighting the fact that incorrect apps may have harmful consequences (Boudreaux et al. 2014; Boulos et al. 2014). However, it should also be noted that no clinician is infallible and that there is also the obvious potential for clinicians to make errors themselves (Iedema et al. 2006). Concerns were also raised by the participants with regard to the personalisation of apps, with respect to specific patients and the relationship between health professionals and their patients. Participants noted that each patient’s clinical presentation is different and that management should, therefore, be patient-specific, which was unlikely to happen with generic apps that aimed to serve a large population. It is important that apps that are prescribed to patients should be flexible enough to suit patients’ specific needs (Ravenek & Alvarez 2016). However, patient-specific apps that provide tailored programmes for their individual needs have not yet been developed (Ernsting et al. 2017).

Participants were also concerned that there might be other barriers to prescribing apps to patients; for example, they noted that older patients might not be open to using apps. This is a valid concern because many health-related apps are not aimed at older populations (Berkowitz et al. 2018). In addition, there is evidence that some older adults may not have the necessary digital literacy to make effective use of apps as part of their management programmes (Ernsting et al. 2017). However, it should also be noted that the use of reminders in the form of text messaging can be useful, especially for older patients, who are more at risk of forgetting about their exercise programmes and appointments (Lilje et al. 2017).

If physiotherapists are going to move in the direction of other health care professionals and begin prescribing and using apps as part of their clinical practice, it is important that they are aware of the many challenges that exist as part of the process, including making choices about the reliability, accuracy and quality of apps, which are mostly unknown (Paglialonga, Lugo & Santoro 2018). For example, the suggestions provided by apps should not be taken at face value, and physiotherapists will need to use their clinical judgement in order to help patients develop a more nuanced understanding of how apps might inform their decision-making. Studies of medical apps and health-related apps have shown that health care providers and users of apps should adopt a critical stance before advocating for their use. Health practitioners need to review apps with regard to their helpfulness, how they function, ease of use and accuracy before prescribing them to their patients (Boudreaux et al. 2014). It is clear that apps have considerable limitations in clinical practice and they may also be costly to implement. Further research that includes clinical trials and observational studies are necessary in order to assess how medical apps and health-related apps are best used in clinical practice (Aitken 2013; Boulos et al. 2014).

Clinicians will need to make sure that their use of apps is tailored to the patients who are most likely going to benefit from their use. While clinicians are busy and may not have enough time and resources to properly assess apps before prescribing them, this is an essential component of ensuring that the apps they suggest are fit for the purpose (Berkowitz et al. 2018). However, our study found that participants lacked the knowledge on how to select the most appropriate apps to use, suggesting that they would require guidance and recommendations when it comes to prescribing apps. Unfortunately, there is very limited evidence with which to inform decision-making when it comes to choosing and using medical apps and health-related apps as part of clinical practice. Studies have identified that health professionals should increase their understanding of apps, and mobile technology in general, and that this should be incorporated into the undergraduate curriculum (Ravenek & Alvarez 2016).

Universities and professional organisations can play a role in educating health professionals on apps and the technology involved. Training and education need to be provided to health professionals, which could equip them to transition app integration into clinical practice (Chen et al. 2017b). Indeed, collaboration amongst all stakeholders is necessary in order to produce improved patient-centred apps (Chen et al. 2017a). It would not be useful to suggest not using apps – as this is unlikely to prevent actual use – but rather suggest that clinicians adopt a critical and zealous approach to their careful use (Abbott & Smith 2018). All stakeholders should play an important role with regard to the proper use of appropriate and high-quality medical apps and health-related apps in clinical practice (Wilhelmina et al. 2016). With an increasing number of students and clinicians using smartphones as part of their learning and professional practice, it could be argued that there is a need to define the scope, and also design and develop suitable guidelines for the use of apps in practice in order to ensure that the work of clinicians is not compromised (Payne, Wharrad & Watts 2012).

## Conclusion

While this study does not attempt to analyse all possible apps in all clinical contexts, it serves as an initial description of an emerging field of clinical practice. The study aimed to identify how a small group of South African physiotherapists made use of smartphone apps as part of their clinical practice. While we acknowledge that the low response rate and the relatively small sample size for the study make it impossible to generalise our findings to a larger population, we nonetheless believe that this study provides a baseline understanding of app use and concerns about practice amongst a small group of South African physiotherapists. Many participants did not prescribe apps to patients and suggested reasons as to why this was the case including their own lack of knowledge on app quality, the availability of apps, their purposes and how they should be used. We also found that clinicians used apps predominantly to augment administrative tasks as part of their practice. Several challenges were highlighted, mainly phrased as concerns around app quality and the responsibility for patient safety. These concerns mainly stemmed from a lack of knowledge of apps and their uses. Educational institutions and professional organisations may need to educate professionals by producing guidelines for the development of apps, as well as giving clinicians a foundation for addressing the challenges raised with their use. Further research is required to guide the way forward in the use of apps in practice. While there is certainly the need for a better understanding of the value that is derived from the use of smartphone apps, the aim of this descriptive study was to identify a baseline profile of app use amongst a small sample of South African physiotherapists. Using these findings should help begin an informed conversation about how South African physiotherapists could think about the use of medical apps and health-related apps as part of clinical practice. The aim of improving patients’ health outcomes is the driving force behind the decisions made by professionals, who will need to make use of their own judgement with respect to deciding who to prescribe apps to, as not all patients would be open to these recommendations. There is also a need for understanding the purpose of apps, as this study identified that most prescribed apps were for exercise programmes, general health and wellness information, and reminders. In other words, contrary to some of the concerns expressed by participants, these apps cannot replace the physiotherapist but rather augment their practice. Thus, the incorporation of mobile technology is not aimed at eliminating health professionals but rather to empower patients, enabling them to take ownership of their own health.
